# Histone H3.3 K27M chromatin functions implicate a network of neurodevelopmental factors including ASCL1 and NEUROD1 in DIPG

**DOI:** 10.1186/s13072-022-00447-6

**Published:** 2022-05-19

**Authors:** Nichole A. Lewis, Rachel Herndon Klein, Cailin Kelly, Jennifer Yee, Paul S. Knoepfler

**Affiliations:** 1 Department of Cell Biology and Human Anatomy, University of California Davis School of Medicine, Sacramento, CA 95817 USA; 2 Genome Center, University of California Davis School of Medicine, Sacramento, CA 95817 USA; 3 Institute of Pediatric Regenerative Medicine, Shriners Hospital for Children Northern California, Sacramento, CA 95817 USA

**Keywords:** ATAC-seq, ASCL1, NEUROD1, diffuse midline glioma, Super-enhancers, Epigenetics, DIPG, H3.3K27M, Open chromatin, Transcription factor binding motifs

## Abstract

**Background:**

The histone variant H3.3 K27M mutation is a defining characteristic of diffuse intrinsic pontine glioma (DIPG)/diffuse midline glioma (DMG). This histone mutation is responsible for major alterations to histone H3 post-translational modification (PTMs) and subsequent aberrant gene expression. However, much less is known about the effect this mutation has on chromatin structure and function, including open versus closed chromatin regions as well as their transcriptomic consequences.

**Results:**

Recently, we developed isogenic CRISPR-edited DIPG cell lines that are wild-type for histone H3.3 that can be compared to their matched K27M lines. Here we show via ATAC-seq analysis that H3.3K27M glioma cells have unique accessible chromatin at regions corresponding to neurogenesis, NOTCH, and neuronal development pathways and associated genes that are overexpressed in H3.3K27M compared to our isogenic wild-type cell line. As to mechanisms, accessible enhancers and super-enhancers corresponding to increased gene expression in H3.3K27M cells were also mapped to genes involved in neurogenesis and NOTCH signaling, suggesting that these pathways are key to DIPG tumor maintenance. Motif analysis implicates specific transcription factors as central to the neuro-oncogenic K27M signaling pathway, in particular, ASCL1 and NEUROD1.

**Conclusions:**

Altogether our findings indicate that H3.3K27M causes chromatin to take on a more accessible configuration at key regulatory regions for NOTCH and neurogenesis genes resulting in increased oncogenic gene expression, which is at least partially reversible upon editing K27M back to wild-type.

**Supplementary Information:**

The online version contains supplementary material available at 10.1186/s13072-022-00447-6.

## Background

Diffuse intrinsic pontine gliomas (DIPG) are a leading cause of cancer-related deaths in children with survival typically being less than 2 years [1]. One of the defining features of DIPG is the presence of the histone tail mutation H3.3K27M, which is estimated to be in about 80% of these tumors, and is now classified by the World Health Organization (WHO) as *diffuse midline glioma, H3 K27M-mutant* [2–5]. The K27M mutation greatly impacts epigenetic modifications  such as causing global decreases in the repressive mark H3K27me3, an increase in the activating mark H3K27ac, and a reduction in DNA methylation [6–9]. Current data suggests that H3.3K27M either sequesters, excludes, or enzymatically inactivates PRC2 thus preventing it from methylating wild-type H3 nucleosomes except at strong affinity sites that lack H3.3K27M deposition [6, 9–17]. A more recent hypothesis is that H3K27M also prevents the spread of H3K27me3 from high-affinity PRC2 sites, specifically large unmethylated CpG islands related to lineage differentiation [18].

The exact scope of oncogenic mechanisms are still to be defined, but it is established that H3.3K27M disruption to epigenetic marks are involved in the maintenance of tumor properties [18, 19]. Several studies have been conducted investigating interventions to correct the disrupted epigenetic landscape with some showing possible clinical promise. Namely, small-molecule inhibitors of EZH2, the catalytic subunit of PRC2 responsible for the H3K27me3 mark, and the histone deacetylase inhibitor (HDACi) panobinostat have been heavily investigated with the latter advancing to clinical trials [14, 15, 20, 21]. Further decreasing H3K27me3 initially appears counter-intuitive, but this approach reduced H3K27me3 at tumor–suppressor genes that are otherwise silenced in DIPG leading to reduced proliferation [14, 15]. Similarly, further increasing histone H3 acetylation has the unexpected result of partially restoring H3K27me3 levels due to histone tail polyacetylation blocking PRC2 and H3K27M interactions leading to decreased proliferation and restoring aspects of normal gene expression [20, 22]. However, both strategies have pitfalls including patient-to-patient variability, development of resistance, and cytotoxicity [14, 15, 20, 23–25]. This complexity demonstrates the necessity to further understand the mechanism(s) at play in DIPG that disrupt histone marks and the downstream effects on chromatin and gene expression to identify other potential drug targets.

Importantly, the H3.3K27M mutation, and subsequent chromatin mark changes, in DIPG results in modifications to the transcriptome that are mostly unique from those associated with H3.1K27M DIPG and H3-WT glioblastoma including increased expression of genes related to neural development, neurogenesis, NOTCH signaling, and differentiation [3, 18, 19, 26, 27]. Drug studies have demonstrated that inhibition of the NOTCH pathway leads to both reduced proliferation and viability in DIPG further supporting its importance to tumor maintenance and presenting a promising component of potential future combinatorial treatment methods [19, 28, 29]. While the epigenetic effects of K27M have been extensively studied, relatively much less is known about the impacts on chromatin structure and function [23, 30].

Here we build upon our previous work, where we used CRISPR–Cas9 to gene-edit the H3.3K27M point mutation in established DIPG lines to generate isogenic H3.3 wild-type cell lines [19]. We assessed differential open chromatin regions (ATAC-seq) between the pairs of isogenic DIPG cell lines and found that in H3.3K27M DIPG cells genes regulating neurogenesis and neuronal processes were enriched in open chromatin regions, including their corresponding enhancers and super-enhancers, and had increased gene expression in H3.3K27M DIPG compared to their matched control H3.3 wild-type gene-edited cell lines. Binding motifs for ASCL1, which we previously showed to be upregulated in H3.3K27M and important for tumorigenic functions, and NEUROD1, a transcription factor known to be essential for normal neurogenesis, were enriched in H3.3K27M cells at genes related to neuronal processes and nervous system development that contained unique open chromatin regions in K27M cells. Based on these findings we propose a model in which H3.3K27M nucleosomes cause a more euchromatic structure at super-enhancers and gene bodies of genes related to neurogenesis and NOTCH signaling, exposing the binding site motifs for key transcription factors, such as ASCL1 and NEUROD1 resulting in increased expression of their target genes and thus contributing to tumorigenesis.

## Results

### K27M-dependent accessible chromatin regions in pediatric gliomas

To study how the oncohistone H3.3K27M affects chromatin dynamics and how chromatin landscape changes impact gene expression we used Assay for Transposase-Accessible Chromatin using sequencing (ATAC-seq) [31–33] on our previously described panel of isogenic DIPG cell lines that were gene-edited to have wild-type H3.3 using CRISPR-Cas9 [19]. Briefly, two H3.3K27M DIPG lines SU-DIPG-XIII and SU-DIPG-XVII (hereafter referred to as XIII and XVII, respectively, and also more generally as “parental” cells), and their CRISPR gene-edited counterparts (XIII-WT and XVII-WT) were used for this study.

**Fig. 1:**
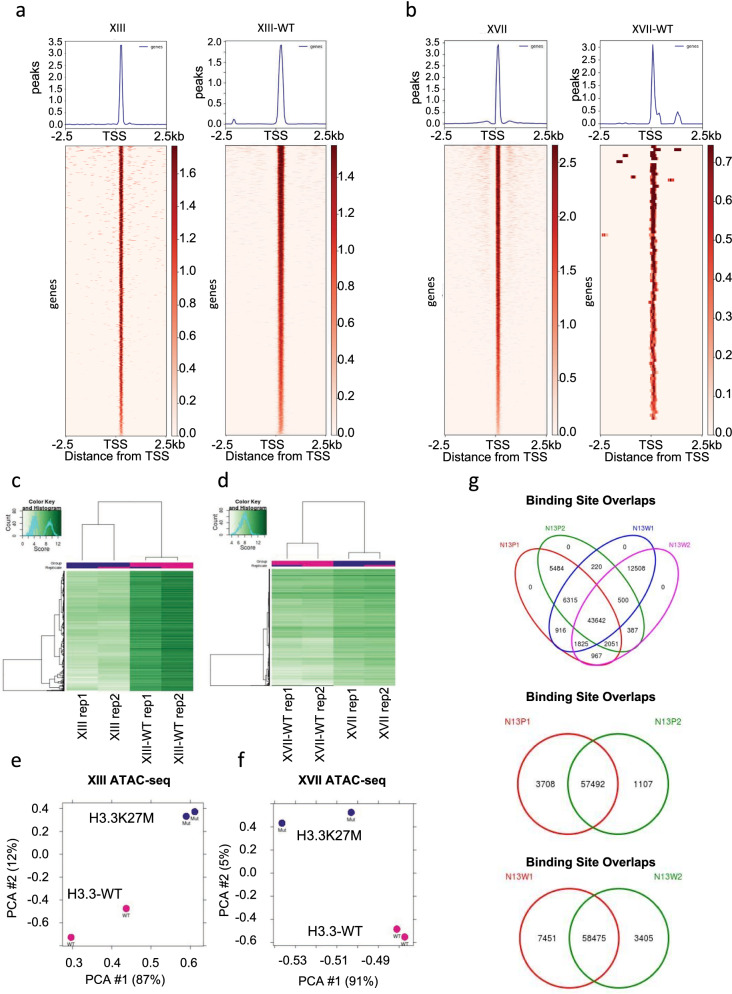
Genome-wide profile of accessible chromatin regions in H3.3K27M and H3.3-WT DIPG tumor samples. **a** Read density heatmaps and average profiles of ATAC-seq peaks for XIII and XIII-WT, **b** XVII and XVII-WT.** c** Hierarchical clustering analysis of accessible chromatin regions via ATAC-seq comparing XIII and XIII-WT, **d** XVII and XVII-WT. **e** Principal component analysis (PCA) of significantly differential peaks between XIII and XIII-WT, **f** XVII and XVII-WT. **g** Overlap of ATAC-seq peaks in XIII and XIII-WT duplicates (top), XIII duplicates alone (middle), and XIII-WT duplicates alone (bottom)

We performed ATAC-seq using biological duplicates for each cell line and after peak calling used the R package DiffBind to identify open chromatin peaks that are differential between H3.3-WT and H3.3K27M cell lines. The XIII and XIII-WT cells had 4522 and 12,860 unique peaks, respectively, and XVII and XVII-WT had 13,136 and 211 unique peaks, respectively (Additional file 1: Table S1). Distinct chromatin accessibility profiles were observed between H3.3-WT and H3.3K27M cells for each line showing that the presence or absence of H3.3K27M substantially changes chromatin accessibility (Fig. 1a–d). Principal component analysis (PCA) of the matched isogenic lines indicated high reproducibility between replicates and that H3.3-WT and H3.3K27M separate from each other (Fig. 1e, f). When both sets of matched isogenic lines were included in the DiffBind analysis hierarchical clustering suggested that lines clustered in part based off *H3F3A* mutation status, that is H3.3K27M lines clustered together, while wild-type H3.3 clustered together (Additional file 1: Fig. S1a). However, PCA analysis also suggested some bias for clustering based on the original cell line with XVII and XVII-WT separating from XIII and XIII-WT (Additional file 1: Fig. S1b). This is further supported by overlapping peaks found in each duplicate of XIII (N13P1 and N13P2) and XIII-WT (N13W1 and N13W2) with most peaks being common to both duplicates of each line (Fig. 1g, middle and bottom Venn diagram). In addition, no peaks were unique to one duplicate and instead all peaks were shared between either one of the duplicates (i.e., N13P1 and N13P2 or N13W1 and N13W2) or with the other cell line (i.e., N13P1 and N13W1) (Fig. 1g, top Venn diagram). This suggests that across DIPG cell lines the presence of H3.3K27M results in significant differences in open chromatin regions compared to wild-type, but there are many unique features in DIPG patient lines that are maintained regardless of *H3F3A* status. In addition, only a small proportion of open chromatin peaks were mapped to regions we previously defined as having differential H3.3 deposition between parental and wild-type lines [19] suggesting that the presence of H3.3 alone does not exclusively drive changes in chromatin accessibility in DIPG (Additional file 1: Table S1). These findings point to other factors likely influencing open chromatin regions that are specific to each cell line.

**Fig. 2:**
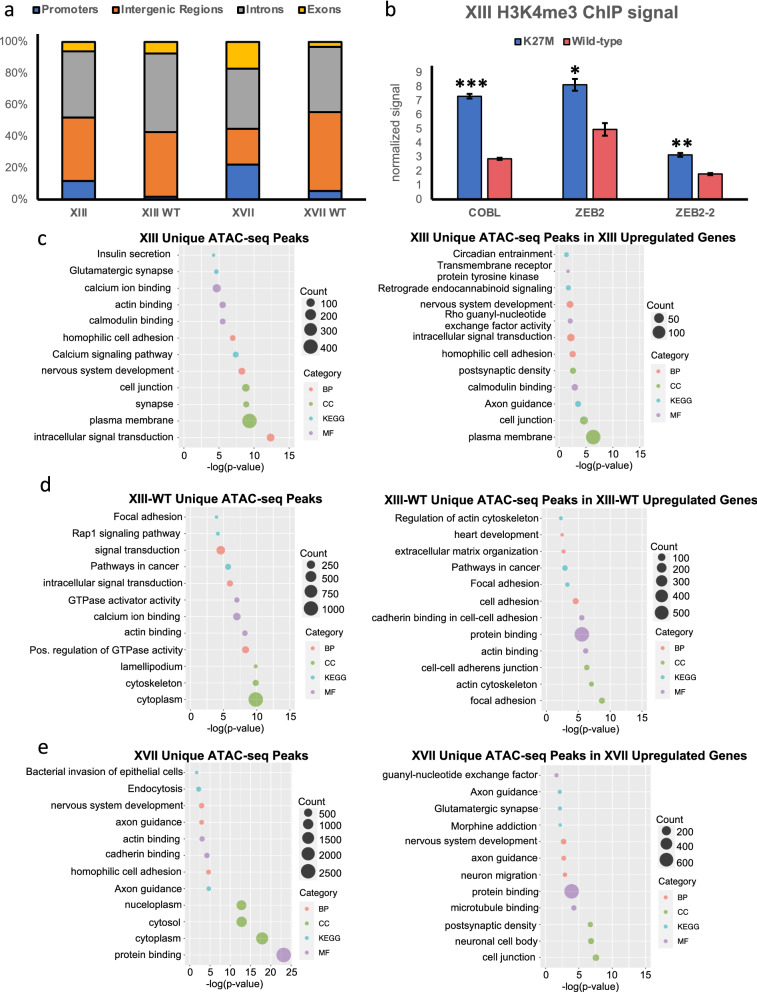
H3.3K27M DIPG cells are enriched for open chromatin peaks in upregulated genes related to the nervous system compared to their isogenic gene-edited wild-type counterparts. **a** Bar plot showing proportion of peaks in various genomic regions. Promoters were defined as 2 kb upstream of the TSS and 1 kb downstream. 75% or more of the peaks for each line were found in intergenic regions or introns. **b** H3K4me3 levels at genes associated with changes in ATAC-seq peaks between Line XIII PAR (K27M, blue) and WT (wild-type, red) cell lines, validated by ChIP–qPCR. *n* = 2 biological replicates, *p value < 0.05, **p value < 0.01, ***p value < 0.001 and error bars were calculated based on standard deviation. **c** Gene ontology analysis of differential open chromatin (left side) and specifically open chromatin regions linked to increased gene expression (right side) in XIII, **d** XIII-WT, and **e** XVII. *BP* biological processes, *CC* cellular components, *KEGG* KEGG Pathways, and *MF* molecular function. For enriched GO terms *p* values were obtained from the Benjamini–Hochberg method

We determined the proportion of accessible chromatin peaks that were in exons, introns, promoters (defined as between 2 kb upstream and 1 kb downstream of the TSS), and intergenic regions of the genome for each cell line. Interestingly, the percentage of accessible peaks mapped to the promoter regions decreased upon reversion of H3.3K27M to wild-type (Fig. 2a). This finding was further supported via H3K4me3 chromatin immunoprecipitation (ChIP) qPCR of two such regions. *COBL* is an actin nucleator involved in  neurite outgrowth and of particular importance to proper cerebellum formation [34, 35] and *ZEB2* is a zinc finger E-box binding transcription factor that plays a role in promoting migration and invasion of both adult and pediatric glioblastoma cells [36, 37]. We demonstrated a significant decrease in H3K4me3 levels upon reversion to wild-type in promoter regions of both genes (Fig. 2b). Approximately 40% or more of the peaks across all lines (except XVII, which had a more even distribution across all genomic categories) were found in intergenic and intronic regions, while the remaining peaks were found in promoters and exons (Fig. 2a and Additional file 1: Table S1). This analysis demonstrates that most of the accessible chromatin is located in regulatory regions, suggesting that changes to gene expression are often due to increased enhancer or super-enhancer activity.

### Open chromatin regions are uniquely enriched for neuronal genes in K27M cells

Within the ATAC-seq data sets, we looked to see which functional groups of genes were enriched in the open chromatin peaks using gene ontology (GO) analysis of biological processes, cellular components, molecular function, and KEGG pathways. Both parental lines (XIII and XVII) showed enrichment for nervous system development, homophilic cell adhesion, and actin binding terms in the genes in their respective open chromatin regions highlighting processes that are likely important to DIPG biology more broadly (Fig. 2c, e, left panels). Due to low peak numbers, no significant GO terms were identified for XVII-WT but the XIII-WT open regions corresponded to GO terms related to cytoskeleton, actin binding, and focal adhesion (Fig. 2d, left panel).

When all four lines of ATAC-seq data were compared using DiffBind some specific gene clusters were enriched in all H3.3K27M cell lines compared to wild-type H3.3 and vice versa. Parental lines XIII and XVII shared 463 peaks, equating to 245 genes, while XIII-WT and XVII-WT shared 3401 peaks and 1378 genes (Additional file 1: Table S1). This resulted in only one significant GO term being shared between parental line peaks (intracellular signal transduction, data not shown), despite sharing the key H3.3K27M mutation that is a defining feature of DIPG, and demonstrating that there is some degree of heterogeneity between patient tumors. The CRISPR gene-edited H3.3 wild-type cells, however, did have significant shared GO terms including cytoskeleton, cytosol, pathways in cancer, and actin binding further supporting the hypothesis that H3K27M plays a key role in regulating chromatin accessibility at genes that contribute to cell morphology and tumor characteristics (Additional file 1: Fig. S2).

We also compared our ATAC-seq data with the 3ʹTag-seq gene expression data from our previous study [19] to see if the differentially open regions of chromatin in each cell line corresponded to specific genes that showed increased expression in the same cell types. ATAC-seq peaks from DiffBind for each cell line were overlapped with the genomic coordinates of genes that demonstrated increased expression via Tag-seq analysis. This intersection analysis determined that 1881 and 5627 statistically significant upregulated genes in XIII and XVII, respectively, mapped to unique open chromatin ATAC-seq peaks in the two parental cell lines (Additional file 1: Table S2) as compared to their respective WT counterparts. Conversely, 4874 and 119 statistically significant upregulated genes in the H3.3-WT cell lines corresponded to open chromatin ATAC-seq peaks in XIII-WT and XVII-WT, respectively (Additional file 1: Table S2). This analysis demonstrated that many genes with increased expression also reside in these unique accessible regions of chromatin, likely explaining in part why these genes are upregulated.

GO analysis was conducted on these overlaps to determine which gene families were enriched for open chromatin possibly resulting in increased expression (Fig. 2c–e, right panels). Parental lines (XIII and XVII) had open chromatin in numerous upregulated genes related to the nervous system development, cell junction, axon guidance, and postsynaptic density (Fig. 2c, e, right panels). These terms were not present in the XIII-WT GO analysis indicating that enrichment of these processes and changes in corresponding genes  are H3.3K27M-dependent and likely play key roles in DIPG disease biology. The XIII-WT line had open regions corresponding to upregulated genes in terms related to adhesion, actin, and the extracellular matrix (Fig. 2d, right panels). This is consistent with our observation that XIII-WT cells undergo morphological changes and become more adherent even in suspension culture conditions following the reversion of H3.3K27M to wild-type [19].

### Enhancer analysis points to nervous system signaling and stem cell pathways

**Fig. 3:**
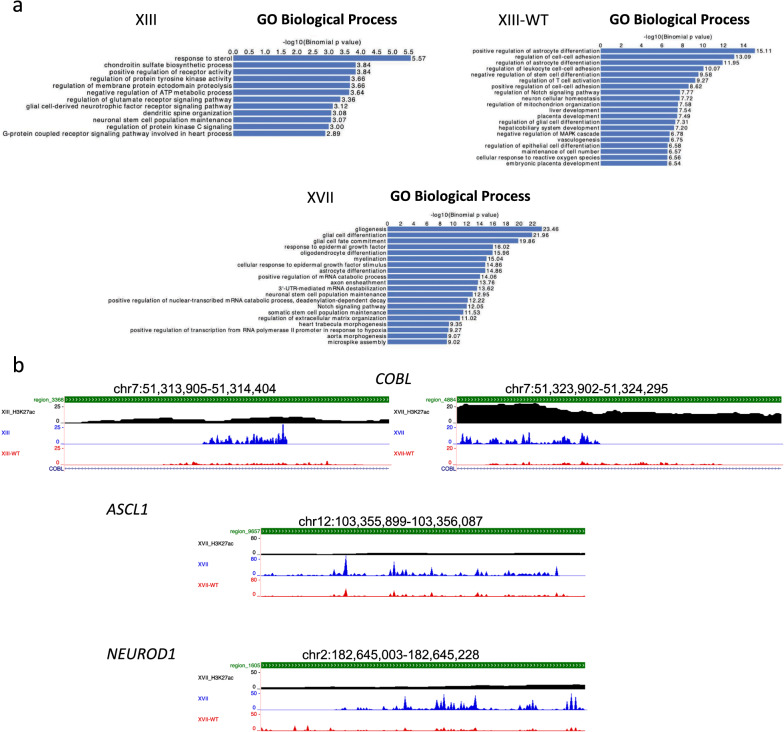
H3.3K27M and H3.3-WT DIPG have differentially accessible enhancer regions. **a** GREAT analysis of differentially open chromatin regions between parental (XIII and XVII) and CRISPR gene-edited isogenic wild-type lines (XIII-WT). **b** Gene tracks including our ATAC-seq peaks (parental=blue, wild-type=red), H3K27ac peaks from Nagaraja et al., 2017 GEO: GSE94259 [27] (black), and enhancer regions defined by GREAT analysis for genes of interest (green bar at the top)

Since H3.3K27M DIPG cells have a distinct active enhancer profile compared to normal pons tissue [38], we compared the enhancer regions with open chromatin specific to our parental DIPG to the isogenic wild-type DIPG lines using the Genomic Regions Enrichment of Annotations Tool (GREAT) analysis (Fig. 3a) [39]. Briefly, enhancers were defined as regions enriched for H3K27ac, the peaks for which were obtained from a previous ChIP-seq data set [27] and were called using MACS2 default parameters [40, 41], excluding promoters as previously defined (Additional file 1: Table S3). Similar to the GO analysis conducted on gene bodies and upregulated genes, parental H3.3K27M lines were enriched for enhancers linked to genes related to various signaling pathways and nervous system development including NOTCH signaling and myelination, both of which have previously been shown to play a role in pons development and be upregulated in DIPG tumors [19, 29, 42]. In addition, neuronal stem cell population maintenance and oligodendrocyte differentiation-related enhancers were present in parental lines supporting findings that key mutations, such as H3.3K27M, likely occur in neural precursor cells (NPCs)/neural stem cells (NSCs) but the cells continue to either partially differentiate into oligodendrocytes or at least gain key oligodendrocyte lineage characteristics and this disrupted development contributes to tumor formation [38, 42–44]. Visualization of ATAC-seq peaks for select genes of interest overlaid with the previously described H3K27ac ChIP-seq data set identified increased accessible chromatin peaks that mirrored increased H3K27ac signal within known enhancer regions for genes knowntobe involved in NOTCH signaling and neuronal development including ASCL1 and NEUROD1 (Fig. 3b).

Conversely, XIII-WT cells continued to show enrichment for processes important to cell adhesion and differentiation into a variety of cell types (Fig. 3a). Interestingly, the GO category negative regulation of MAPK cascade, which has been shown to be an important signaling pathway in DIPG tumors, was also enriched in XIII-WT enhancers, demonstrating that reverting the K27M mutation may be sufficient to somewhat reverse the activation of this pathway and contribute to a decrease in tumor-like characteristics in the isogenic line [27, 38]. Together these results suggest that K27M specifically contributes to chromatin accessibility at enhancers that are important to cell identity and key signaling pathways resulting in activation of processes linked to DIPG tumorigenesis.

### Open chromatin regions are enriched for transcription factor binding sites related to neuronal lineage in K27M cells

**Fig. 4:**
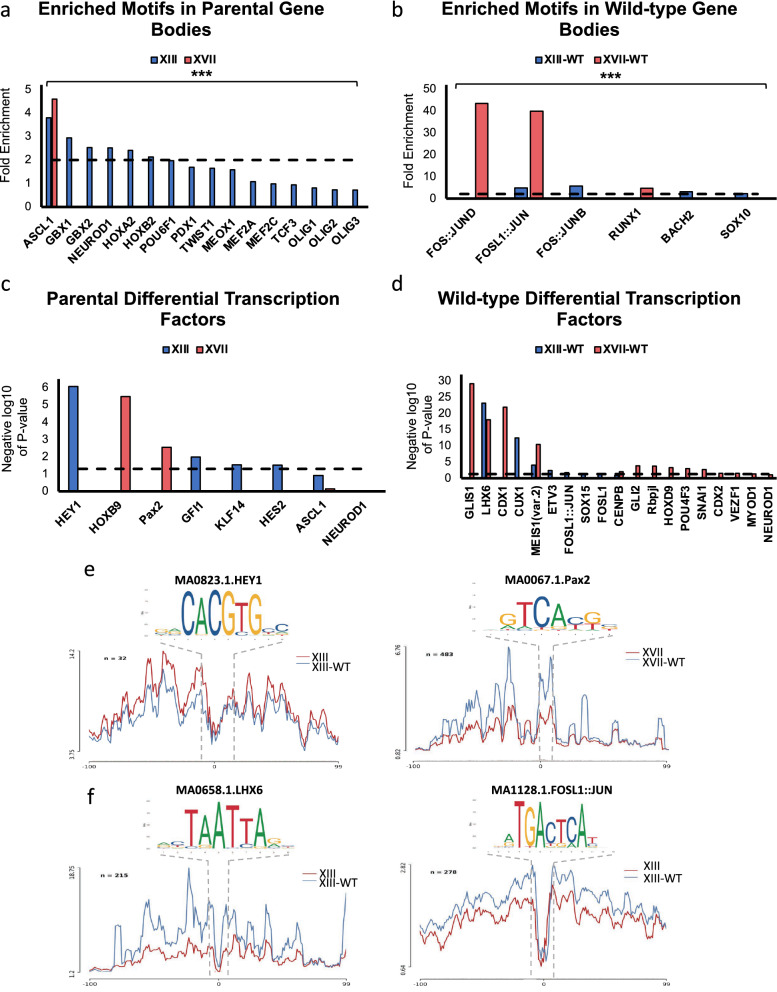
Determination of physiologically relevant enriched transcription factors in H3.3K27M and H3.3 wild-type cells. **a**, **b** Motifs identified using MEME–ChIP and number of times that motif occurred in the ATAC-seq peaks was determined using FIMO. Background was calculated by scrambling DNA sequence and inputting that sequence into FIMO. Threshold for significant fold enrichment was set to 2 (black dashed line). ***p value < 0.001 as described in MEME–ChIP output. **c**, **d** HINT–ATAC identified DNA binding footprints in ATAC-seq data made by transcription factors. Those that were statistically significant (*p* value < 0.05, black dashed line) and of interest from MEME–ChIP analysis are plotted for **c** parental and **d** wild-type. **e** HINT–ATAC line plots showing the differential footprints of transcription factors significantly differentially bound in XIII (HEY1) and XVII (Pax2), and **f** XIII-WT (LHX6 and FOSL1::JUN)

Given the distinct ATAC-seq profiles in gene bodies, enhancers, and super-enhancers, and transcriptomic differences between parental and wild-type cell lines, we also assessed the potential transcription factors at play at the interface between gene expression, chromatin, and regulatory elements. We first scanned gene bodies for motifs, as these regions were most notably enriched for transcription factor motifs over promoters and enhancers, and mapped them to parental or wild-type ATAC-seq peak regions using MEME–FIMO and HOMER (Fig. 4a, b and Additional file 1: Fig. S3a, b). One notable finding was enrichment of the DNA binding motif for ASCL1*,* which we previously identified as upregulated in H3.3K27M DIPG cells and important for their cancer-related cellular functions [19], in both XIII and XVII cells compared to their isogenic wild-type counterparts. Motifs for transcription factors known to play roles in stem cell potency, development, and differentiation into the neuronal lineage including *GBX1, GBX2, NEUROD1, OLIG2,* and *HOXA2* were also enriched in parental K27M cells (Fig. 4a and Additional file 1: Fig. S3a). In support of previous data, GO analysis of *ASCL1* and *NEUROD1* FIMO regions indicated that these motifs were found in genes related to nervous system development, axon guidance, GTPase activity, cell junction, and postsynaptic density (Additional file 1: Fig. S3c, d). Notably, we performed the same motif analysis using MEME–ChIP with peaks that were found to be shared by XIII and XIII-WT to determine motifs that are conserved across glioma independent of H3K27M status. We found that peaks shared between parental and H3.3 wild-type were enriched for *KLF15, ASCL1, NEUROD1*, and *NFYA* transcription factors (Additional file 1: Fig. S4). By contrast, XIII-WT and XVII-WT cells were enriched for a number of FOS::JUN family transcription factors motifs, which have previously been identified to either be enriched in wild-type H3K27 high-grade gliomas or shared across multiple glioma subtypes (Fig. 4b and Additional file 1: Fig. S3b) [23].

We used HINT–ATAC [45] to identify candidate differential transcription factor footprints in open chromatin regions of parental and wild-type cell lines. This analysis also identified transcription factors related to NOTCH signaling, neurogenesis, development, and oncogenesis including *HEY1, PAX2,* and *HOXB9* in parental lines (Fig. 4c, e). While the MEME–FIMO and HINT–ATAC analyses did not identify shared specific factors in K27M lines, the factors that were identified did belong to shared pathways further highlighting the importance of these pathways in DIPG disease biology. *FOSL1::JUN* was present in wild-type cells using both analysis methods as well as factors related to migration, cytoskeleton remodeling, and Sonic Hedgehog signaling including *LHX6, GLI2, CDX1, CDX2,* and *SNAI1* (Fig. 4d, f, Additional file 1: Fig. S3e). This is consistent with GO analysis of XIII-WT ATAC-seq peaks and open chromatin corresponding to increased gene expression (Fig. 2d). Thus, transcription factor analysis further confirmed differences between parental and wild-type lines that are H3.3K27M-dependent and likely driven by altered functions of neuronal factors and NOTCH signaling.

**Fig. 5:**
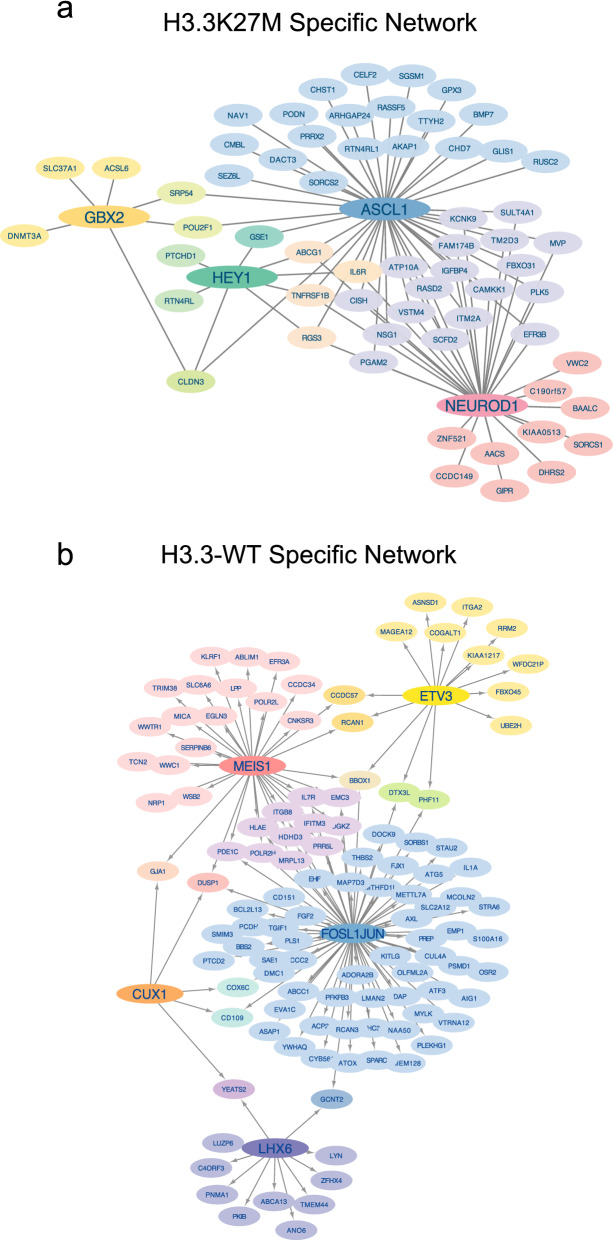
Network analysis indicates that ASCL1 and NEUROD1 have overlapping targets specifically in K27M DIPG. **a** H3.3 K27M-specific gene regulatory network in DIPG showing enriched motifs in K27M specific ATAC-seq peaks at genes also upregulated in K27M DIPG cells compared to WT DIPG cells. **b** H3.3 WT-specific gene regulatory network in DIPG showing enriched motifs in WT specific ATAC-seq peaks at genes upregulated in WT DIPG cells compared to their isogenic K27M DIPG counterpart cells

We next sought to understand the K27M-specific transcriptional regulatory relationships through network analysis. We used FIMO to scan the K27M-specific ATAC-seq peaks at upregulated genes for the motifs identified as enriched in the HOMER and HINT–ATAC motif analysis and then used Cytoscape to build a network of potential transcriptional regulation (Fig. 5). Our analysis revealed a striking overlap between ASCL1 and NEUROD1 motifs in these regions, suggesting the two factors may act coordinately to activate a K27M-specific oncogenic gene network (Fig. 5a). Due to this overlap we investigated if ASCL1 and NEUROD1 physically bind together using co-immunoprecipitation, but no binding was detected (data not shown). We repeated the functional analysis for WT cells and the resulting network suggests that FOSL1::JUN is a major hub for regulating H3.3 WT specific DIPG gene expression (Fig. 5b). There is also a substantial overlap between FOSL1::JUN and MEIS1 potential targets, suggesting these two factors may work together or in a pathway to coordinate gene expression. Notably, LHX6 appears to primarily regulate a putative set of targets distinct from the other identified factors, while CUX1 motifs are all found with the motifs of additional factors, pointing to a more cooperative role in regulation.

### Super-enhancer analysis points to specific neuronal genes in DIPGs

**Fig. 6:**
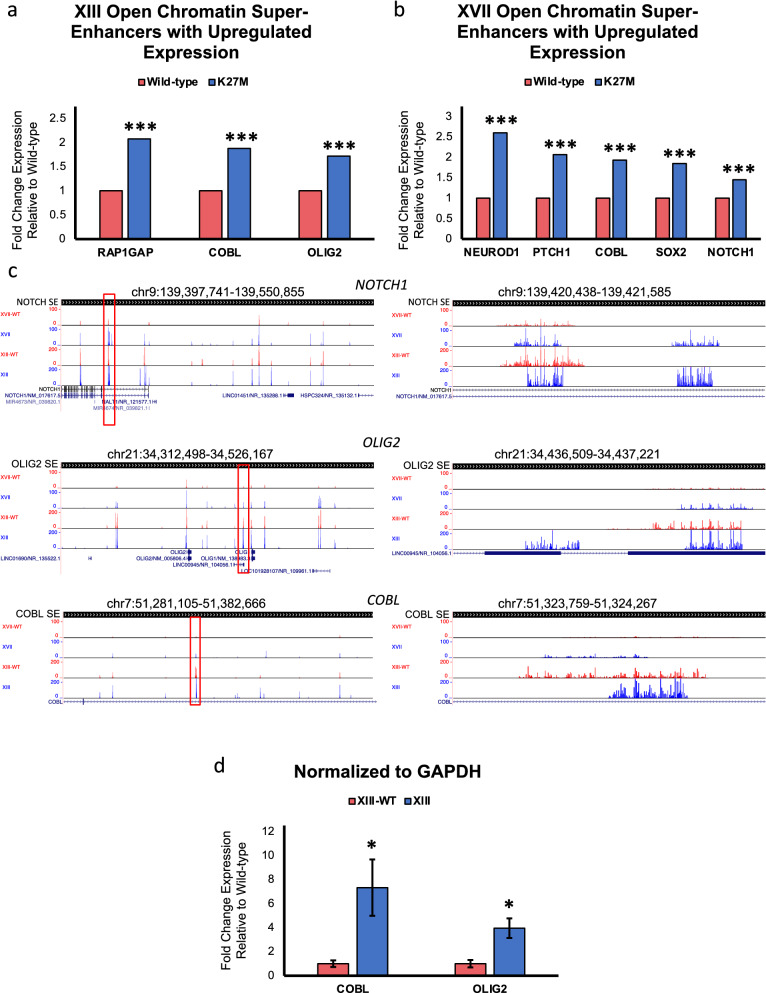
H3.3K27M DIPG cells have accessible super-enhancers linked to increased gene expression compared to isogenic H3.3-WT DIPG samples. **a** Select super-enhancers of interest with open chromatin according to ATAC-seq data and increased gene expression via Tag-seq ranked according to fold change relative to H3.3-WT in XIII and **b** XVII ***p value < 0.001. **c** Gene tracks with ATAC-seq peaks from UCSC Genome Browser of select super-enhancer regions of interest. **d** *COBL* and *OLIG2* were selected for validation using qPCR. Fold change expression in H3.3K27M DIPG were calculated relative to H3.3-WT. *n* = 4, *p value < 0.05, and error bars were calculated based on SEM

Super-enhancers are linked to oncogenesis in many tumor types, including DIPG, and we have previously demonstrated that H3.3K27M leads to loss of H3K27me3 at super-enhancers, resulting in increased expression of linked genes that likely contribute to tumorigenesis [19, 27, 46]. We overlapped our accessible chromatin peaks with previously defined super-enhancers [27] and found that XIII cells had 306 uniquely accessible ATAC-seq peaks within these super-enhancers and 107 of these super-enhancers with K27M-specific ATAC-seq peaks were linked to transcriptionally upregulated genes (Additional file 2: Table S4A). Similarly, XVII cells had 294 super-enhancers with ATAC-seq peaks unique to H3.3K27M, and 99 of these were tied to upregulated genes (Additional file 2: Table S4A). XIII-WT cells showed changes in accessibility at 282 super-enhancers and only 52 of those corresponded to upregulated genes in that cell line (Additional file 2: Table S4B). The XVII-WT cells only had one accessible super-enhancer that corresponded to upregulated genes in that cell line, likely due to the low number of unique peaks, and were not analyzed further (Additional file 2: Table S4B). In addition, visualization of ATAC-seq peaks for select super-enhancer regions of interest displayed differences in peak patterns between parental and wild-type cell lines providing further evidence that H3.3K27M deposition results in more open chromatin, especially at key regulatory regions, such as super-enhancers (Fig. 6c).

Upregulated genes linked to super-enhancers in either parental line included several related to neurogenesis and NOTCH signaling including *NOTCH1, RAP1GAP, POU3F2,* and *NEUROD1* as well as genes important for stemness, differentiation, and development, such as *SOX2, OLIG2,* and *PTCH1* (Fig. 6a, b and Additional file 2: Table S4A). Further, XIII and XVII cells shared the following nine genes that were associated with accessible super-enhancers and displayed increased expression: *CELF2, COBL, DAB2IP, FAM222A, GAB2, LINC01158, SASH1, STK32B,* and *TSHZ1* (Fig. 6a–c and Additional file 2: Table S4A). From this list of common genes we decided to validate the gene expression of *COBL* via RT-qPCR and also included *OLIG2* as a control due to its established importance in DIPG biology and having already been validated in our previous study [3, 19, 27, 30, 38, 42–44, 47]. We also chose to validate *ZEB2* expression, because we previously showed it to be upregulated in XIII and XVII [19] and H3K4me3 levels at the promoter are impacted by H3.3K27M (Fig. 2b). We were able to validate the general changes in gene expression by RT-qPCR (Fig. 6d and Additional file 1: Fig. S5). Taken together, this analysis suggests that the super-enhancers of specific genes become more open in H3.3K27M cells and this chromatin mechanism contributes to aberrant gene expression, whereas in H3.3 wild-type these regions become more closed resulting in a gene expression profile more typical of normal cells.

## Discussion

Major progress has been made in understanding the mechanisms of DIPG biology, with arguably one of the most important discoveries being the prevalence of the mutations in histone H3 proteins, especially in H3.3. The subsequent changes to epigenetic marks and the transcriptome have been extensively examined; however, the more specific mechanisms behind these changes remain an open area of study. In an effort to better understand the machinery behind these epigenomic changes, a few studies have investigated the chromatin landscape in H3.3K27M DIPG and H3.3K27M knockout DIPG cell lines [23, 30]. However, this work did not primarily focus on chromatin accessibility leaving this an under-studied component of DIPG biology. We took this work further using ATAC-seq on our isogenic CRISPR–Cas9 reverted to wild-type H3.3 and H3.3K27M DIPG lines.

To address if more accessible chromatin is in part responsible for increased gene expression we intersected our ATAC-seq data with our previous 3ʹ Tag-seq expression data [19]. GO analysis revealed that XIII and XVII open chromatin regions corresponding to increased gene expression were enriched for terms related to neuronal development. The abundance of nervous system-related terms likely in part reflects the developmental origins of these tumors [38, 47, 48] and also supports the hypothesis that the formation of these DIPG tumors occurs in part through the dysregulation of very specific neuronal developmental programs [14, 18, 44, 48–50]. Conversely, analysis of XIII-WT specific overexpressed genes with open chromatin regions revealed enrichment for morphology genes including actin cytoskeleton and cell adhesion, with little to no enrichment for neuronal terms. Together these results suggest that H3.3K27M results in open chromatin and subsequent increased expression of defined genes related to nervous system development thus driving the progression of tumor formation, while the presence of wild-type H3.3 instead of K27M in DIPG results in open chromatin and upregulation of cell adhesion genes leading to decreases in tumor-like morphology. This is consistent with changes to cell morphology in DIPG cells gene-edited from K27M to a wild-type *H3F3A* state that we had previously observed as well as previous reports indicating that H3K27M DIPG cells express mesenchymal and oligodendroglial gene signatures and phenotypes [19, 25, 44, 51, 52].

A similar pattern was also observed following overlaying the regulatory enhancer and super-enhancer regions with the open chromatin regions that we identified. XIII and XVII cells were enriched for enhancers and super-enhancers related to NOTCH signaling and nervous system development genes including, but not limited to, *ASCL1* and *NEUROD1*, which are basic-helix–loop–helix (bHLH) transcription factors with known functions in neuronal cell fate and differentiation of glioblastoma stem cells (GSCs) as well as normal neuronal cells [53–57]. Our group and others have shown that ASCL1, in connection to NOTCH and WNT signaling pathways, likely plays a key role in GBM and DIPG tumors and can act as a pioneer factor to bind to chromatin to promote a more open chromatin configuration at enhancers of neuronal genes [19, 30, 55, 56, 58]. In medulloblastoma NEUROD1 has a similar role to ASCL1 and has also been shown to be a pioneer factor in embryonic stem cells (ESCs), where it binds to heterochromatic promoters and inactive enhancers resulting in increased H3K27ac levels and subsequent increased expression of neurodevelopment genes [59, 60].

Fitting with our GO results, motif analysis revealed that open chromatin regions in H3.3K27M cells are enriched for ASCL1 and NEUROD1 binding motifs. Those motifs are located in genes related to neuronal development including *COBL,* which is upregulated and has accessible enhancer and super-enhancer regions in H3.3K27M cells. *COBL* is a calcium and calmodulin-dependent actin nucleator in developing neurons, particularly in the cerebellum, that increases neurite formation and branching [34, 35, 61]. Calcium and calmodulin GO terms are enriched in H3.3K27M DIPG open chromatin regions, which could contribute to the aforementioned cell morphology and adhesion changes between our H3.3K27M and H3.3 wild-type cells. It is worth noting that ASCL1 and NEUROD1 motifs were detected and shown to be enriched in peaks shared between parental and H3.3 wild-type lines suggesting that ASCL1 and NEUROD1 have important roles in DIPG beyond H3K27M functions. However, since both ASCL1 and NEUROD1 motifs were enriched in parental H3.3K27M cells over those reverted to wild-type, these transcription factors still likely have unique roles in H3K27M tumors.

While there were consistencies between the two parental K27M DIPG cell lines and separately between the two isogenic H3.3 wild-type lines, some distinct differences remained even between cells of like *H3F3A* status resulting in some degree of sample clustering by principal component analysis based on patient origin rather than completely based on *H3F3A* status. It has been extensively documented that while histone H3 and IDH1 mutation status are the main identifiers for pediatric high grade-glioma (HGG) and DIPG subgroups there are other secondary mutations within these subgroups that drive disease progression [1, 3, 51, 62–65]. Therefore, it is possible that these secondary mutations are involved in chromatin dynamics.

**Fig. 7:**
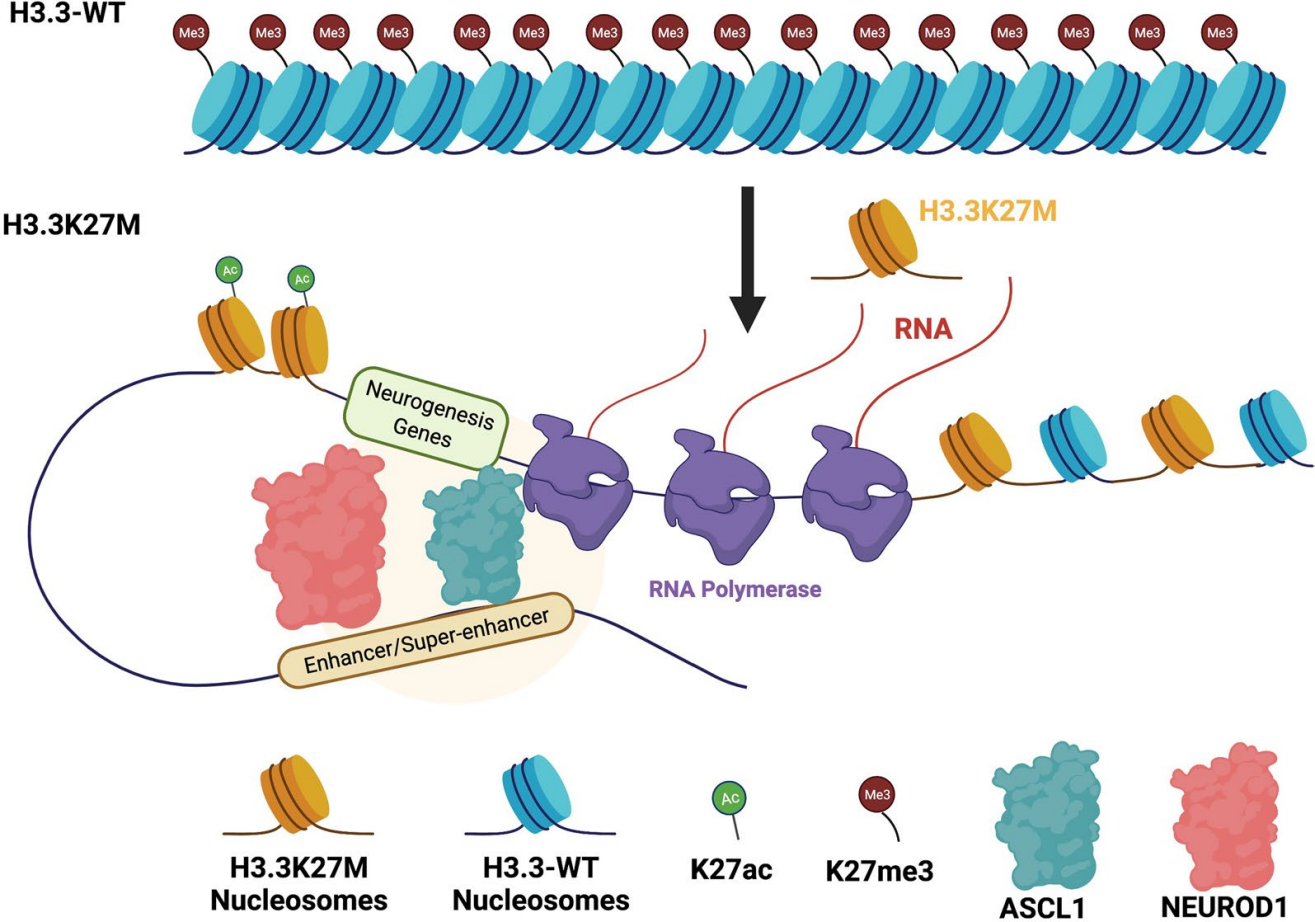
Model of how H3.3K27M affects the chromatin landscape and subsequent gene expression. In this model, we predict that normal brain cells have H3K27me3 in specific regions throughout the genome resulting in heterochromatin or discrete domains of repressed chromatin and decreased gene expression of specific neuronal, NOTCH signaling, and oncogenesis genes. After the introduction of the point mutation in *H3F3A* resulting in the H3.3K27M mutation, H3K27me3 levels are decreased and H3K27ac levels increase. The chromatin begins to aberrantly open upon deposition of H3.3K27M–H3K27ac nucleosomes at defined enhancers and super-enhancers responsible for regulating expression of neurogenesis genes. *ASCL1* and *NEUROD1* transcription factors bind to their respective motifs in these opening regions. We propose that *ASCL1* and *NEUROD1* binding facilitates enhancer and super-enhancer functions and further opens the chromatin regions ultimately resulting in increased expression of specific neurogenesis, NOTCH, and oncogenesis genes (Adapted from “Regulation of Transcription in Eukaryotic Cells”, by BioRender.com (2021). Retrieved from https://app.biorender.com/biorender-templates)

Our study identified an increase in open chromatin in the *OLIG2* enhancer region for both H3.3K27M DIPG lines compared to matched isogenic H3.3 wild-type lines, consistent with a previous study [30], as well as open chromatin in the super-enhancer of *OLIG2* in line XIII and its promoter in line XVII. *OLIG2*, like *ASCL1* and *NEUROD1,* is a basic helix–loop–helix transcription factor and a marker of the oligodendrocyte lineage, one of the proposed cells of origin for DIPG [42, 43, 47], and it has increased expression in H3.3K27M DIPG [19, 48]. Expression of *OLIG2* has been shown to be necessary for DIPG cell tumorigenesis and proliferation within orthotopic mouse xenograft models containing *OLIG2* knockdown demonstrating reduced tumorigenesis and increased mouse survival [66]. This suggests that not only does *OLIG2* expression mark the potential cell population of origin but also presents a potential drug target for DIPGs [66]. In addition, *OLIG2* is an upstream activator of *ZEB2* and increased *OLIG2* levels have been shown to activate *ZEB2* during development leading to oligodendrocyte precursor cell (OPC) maturation and myelination [67]. *ZEB2* is an established regulator of EMT and has been linked to increased invasiveness and migration, poor prognosis for glioma patients, and is upregulated in glioblastoma and in DIPG as demonstrated in this study [36, 37, 68]. Interestingly, *ZEB2* is also linked to Notch signaling during Schwann cell development and acts as a repressor of Notch-Hey2 signaling [69]. While our data shows NOTCH signaling to be upregulated in DIPG this does not rule out the possibility that *ZEB2* may be interacting with NOTCH or one of its downstream targets in a way that drives disease progression in DIPG. Further investigation of *ZEB2* in DIPG and its role in NOTCH signaling could help inform new therapies, especially those that target EMT. Our model suggests that H3.3K27M results in decreased H3K27me3 at the *OLIG2* enhancer ultimately resulting in increased expression of *OLIG2* and driving disease progression (Fig. 7).

Similar to previous work, we found that approximately 50% of our ATAC-seq peaks in H3.3K27M lines had the activating mark H3K27ac [23]. H3K27ac levels are influenced by and in some cases dependent on histone H3 mutation status much like the repressive mark H3K27me3. In DIPG, H3K27ac is often found in heterotypic nucleosomes (one histone H3 being wild-type and the other being H3.3K27M) [9, 15, 30] and is hypothesized to contribute to blocking PRC2 from depositing H3K27me3 resulting in increased gene expression due to increased chromatin accessibility, especially at enhancers and super-enhancers [9, 19, 23, 26, 38].

Future work to clarify these epigenetic mechanisms may involve analysis of chromatin dynamics following drug treatments, including NOTCH inhibition given its promise as a drug target and our data supporting the importance of NOTCH in DIPG biology. Such studies could provide further insights into how drug treatments are affecting open versus closed chromatin and the subsequent changes to gene expression and downstream processes [19, 28, 29], potentially pointing the way to new therapies.

## Conclusions

Taken together, our data support a model (Fig. 7), wherein H3.3K27M nucleosomes are enriched in the genome specifically at enhancers and super-enhancers related to neuronal fate genes that in H3.3 wild-type cells are located in repressed regions containing H3K27me3 [9, 15]. This K27M incorporation in turn begins to open the chromatin and leads to the initial increase in expression of ASCL1 and NEUROD1. These transcription factors then bind motifs within enhancers and super-enhancers to further increase chromatin accessibility and ultimately promote enhancer/super-enhancer interactions to aberrantly induce other neurogenesis and oncogenesis related genes, such as *COBL*. Ultimately, these changes along with other mutations together contribute to glioma formation.

## Methods

### Cell culture

Isogenic H3 wild-type cell lines were made as previously described [19]. All cell lines were cultured in Tumor Stem Media as described in [27]. Briefly, the media contains DMEM/F12 1:1 (Invitrogen), Neurobasal-A (Invitrogen), 10 mM HEPES (Invitrogen), 1× MEM sodium pyruvate (Invitrogen), human basic fibroblast growth factor and human epidermal growth factor (20 ng/mL each) (Shenandoah), human platelet-derived growth factor (PDGF)-A and PDGF-B (20 ng/mL) (Shendandoah), heparin (10 ng/mL) (StemCell Technologies), and B27 without Vitamin A (Invitrogen).

### Assay for transposase-accessible chromatin (ATAC-seq) library preparation

ATAC-seq for cell lines was performed similarly to previously published protocols [31, 33] with some modifications. Cells were dissociated and 100,000 cells were washed twice with cold PBS at 4 °C. Cells were resuspended in 50 uL of ATAC-Resuspension Buffer (10 mM Tris-HCl pH 7.4, 10 mM NaCl, 3 mM MgCl_2_, 0.1% Igepal, 0.1% Tween-20, and 0.01% Digitonin) and triturated until cells were lysed. Samples were incubated on ice for 3 min, washed with 1 mL of the buffer (excluding 0.1% Igepal), and centrifuged at 500 RCF for 10 min at 4 °C. The pellet was resuspended in 50 uL of transposition mixture containing 25 uL of 2× TD Buffer (20 mM Tris-HCl pH 7.6, 10 mM MgCl_2_, 20% Dimethyl Formamide), 5 uL Transposase (Illumina Nextera Kit), 0.5 uL 0.1% digitonin, 0.5 uL 10% Tween-20, and PBS, and incubated at 37 °C for 60 min, and DNA recovered using Zymo DNA Clean and Concentrator-5 Kit. Libraries were generated by PCR in 50 uL reactions (20 uL of samples, 25 uL 2× NEBNext Master Mix, and 2.5 uL of each custom primers made by Integrated DNA Technologies (IDT) (for sequences see [32]). The PCR reaction was as follows: 72 °C for 5 min, 98 °C for 30 s, and 5 cycles of 98 °C for 10 s, 63 °C for 30 s, and 72 °C for 1 min. DNA was recovered using Zymo DNA Clean and Concentrator-5 Kit.

### Bioinformatics

ATAC-seq samples were sequenced in duplicate using the HiSeq with paired-end 150 bp sequencing. Adaptors were removed from raw paired-end sequencing files using bbduk (BBMap version 38.70 BBDuk) [70]. Reads were aligned to the hg19 genome using Bowtie2 (version 1.1.2) [71]. The resultant Sequence Alignment Map (SAM) files were compressed to the Binary Alignment Map (BAM) files on which mitochondrial reads were removed using samtools (samtools 1.4) [72]. Peaks were called using HOMER (v4.11) [73]. Unique peaks in each cell line were identified using the R package DiffBind (R version 3.6.3, DiffBind version 2.12.0) [74, 75]. Genes were defined using the UCSC Main table browser using the following settling: Clade: Mammal, Genome: Human, Assembly: Feb. 2009 (GRCh37/hg19), Group: Genes and Gene Predictions, Track: NCBI RefSeq, Table: UCSC RefSeq (refGene), and Region: genome. These defined genes were then overlapped with the ATAC-seq peaks using bedtools Intersect intervals (Galaxy version 2.30.0) [76] on the public server *usegalaxy.com* to define the gene coordinates of the peaks [77]. Gene ontology analysis was performed using DAVID and GREAT [39, 78, 79]. Motif analysis was performed using MEME–ChIP and FIMO (versions 5.4.1) [80, 81] and HOMER (v4.11) [73]. Modeling of DNA footprinting in ATAC-seq peaks was performed using HINT–ATAC (v0.13.2) [45].

### Network analysis

To identify a K27M-specific transcriptional network, accessible chromatin regions in line XIII Parental cells, were intersected with genes significantly upregulated in line XIII parental (K27M) cells compared to WT cells. FIMO was used to scan these regions for the motifs identified as enriched in K27M ATAC-seq peaks (version 5.4.1) [80, 81]. A network was built based on this analysis and visualized with Cytoscape version 3.9.0 [82]. To identify an H3.3 WT-specific transcriptional network, open chromatin regions in line XIII-WT cells were intersected with genes significantly upregulated in line XIII-WT cells and scanned with FIMO for motifs enriched in WT ATAC-seq peaks.

### ChIP-qPCR

In performing ChIP, cells were crosslinked with 1% formaldehyde, lysed, and sonicated using a Bioruptor Pico (Diagenode) to generate chromatin fragments < 500 bp. For each ChIP, 20–30 μg of sonicated chromatin was used and immunoprecipitated using an H3K4me3 antibody (Millipore 04-745), on magnetic Dynabeads (Invitrogen). For spike-in normalized ChIP-qPCR, 5 μg of sonicated Drosophila chromatin was added to each ChIP sample prior to immunoprecipitation. qPCR enrichment was normalized to Drosophila values across all samples. Primers are listed (5ʹ-to-3ʹ) as follows:

**Table:** 

COBL forward	AAGGACGCCTGCATACAAAC
COBL reverse	GTAGTGGTGGAGCAGGTGGT
ZEB2 forward	AGTTTTGGCCAGAAATGGTG
ZEB2 reverse	GAGTGGCCGAAAGAGATCAG
ZEB2-2 forward	CCCTTTCCTTCGAAAAGTCC
ZEB2-2 reverse	TTGTTTCCTCTGGGAATTGG

### Reverse transcription PCR

RNA was extracted from cells using the NucleoSpin RNA Kit (Macherey–Nagel) from which cDNA was made using the iScript cDNA Synthesis Kit (Bio-Rad). RT-qPCR was performed using the PowerUp SYBR Green Master Mix (Applied Biosystems; for human *COBL* and *OLIG2*, normalized to GAPDH) on a Stratagene Mx3005P. Primers are listed (5ʹ-to-3ʹ) as follows:

**Table:** 

COBL forward	TCGCAGCAGAACTTGGTTCG
COBL reverse	GCATGGCTCCCATTGAGCA
OLIG2 forward	TGGCTTCAAGTCATCCTCGTC
OLIG2 reverse	ATGGCGATGTTGAGGTCGTG
ZEB2 forward	CAAGAGGCGCAAACAAGC
ZEB2 reverse	CCACTCCACCCTCCCTTATTTC
GAPDH forward	GGAGCGAGATCCCTCCAAAAT
GAPDH reverse	GGCTGTTGTCATACTTCTCATGG

### Statistical analysis

For statistics comparing RNA and histone mark levels of specific genes in parental versus wild-type lines by qPCR and ChIP–qPCR (respectively), Student’s *t*-tests with a minimum of *n* = 2 biological replicates were performed using GraphPad *t-*test calculator. Error bars represent s.e.m. or standard deviation as specified in the figure legends. ATAC-seq statistics were performed with the program DiffBind in R. The Benjamini adjusted *p* value was used to determine significance for gene ontology analysis. Otherwise the standard *p* value was used.

## Supplementary Information

**Additional file 1: Figure S1.** Genome-wide profile of accessible chromatin regions in H3.3K27M and H3.3-WT DIPG tumor samples. **Table S1.** Summary of HOMER and DiffBind results for ATAC-seq samples. **Figure S2.** H3.3K27M DIPG cells are enriched for open chromatin peaks genes related to the nervous system and GTPase activity compared to their isogenic gene-edited wild-type counterparts. **Table S2.** Overlap of gene expression changes and ATAC-seq peak changes between WT and K27M cells. **Table S3.** Overlap of enhancer and super-enhancer regions with ATAC-seq peaks in XIII and XVII cell lines. **Figure S3.** H3.3K27M DIPGs specific transcription factor binding sites are enriched in nervous system and neuronal development genes. **Figure S4.** Determination of enriched transcription factors shared between H3.3K27M and H3.3-WT peaks. **Figure S5.** H3.3K27M DIPG cells differentially express transcription factor ZEB2.

**Additional file 2: Table S4.** A. Super-enhancers with open-chromatin and increased gene expression in parental DIPG lines (3ʹ Tag-seq data produced by Chen et al. [19] and super-enhancer data produced by Nagaraja et al. [27]). B. Super-enhancers with open-chromatin and increased gene expression in isogenic wild-type lines (3ʹ Tag-seq data produced by Chen et al. [19] and super-enhancer data produced by Nagaraja et al. [27]).

## Data Availability

ATAC-seq data submission to GEO initiated.
