# Supplementary material for: Histone H3.3 K27M chromatin functions implicate a network of neurodevelopmental factors including ASCL1 and NEUROD1 in DIPG
Source: Epigenetics Chromatin. 2022 May 19;15:18. doi: 10.1186/s13072-022-00447-6 (PMC9121554; doi:10.1186/s13072-022-00447-6)
Supplement: Supplementary file 1 — Additional file 1: Figure S1. Genome-wide profile of accessible chromatin regions in H3.3K27M and H3.3-WT DIPG tumor samples. Table S1. Summary of HOMER and DiffBind results for ATAC-seq samples. Figure S2. H3.3K27M DIPG cells are enriched for open chromatin peaks genes related to the nervous system and GTPase activity compared to their isogenic gene-edited wild-type counterparts. Table S2. Overlap of gene expression changes and ATAC-seq peak changes between WT and K27M cells. Table S3. Overlap of enhancer and super-enhancer regions with ATAC-seq peaks in XIII and XVII cell lines. Figure S3. H3.3K27M DIPGs specific transcription factor binding sites are enriched in nervous system and neuronal development genes. Figure S4. Determination of enriched transcription factors shared between H3.3K27M and H3.3-WT peaks. Figure S5. H3.3K27M DIPG cells differentially express transcription factor ZEB2. [file 13072_2022_447_MOESM1_ESM.docx]

**Histone H3.3 K27M chromatin functions implicate a network of neurodevelopmental factors including ASCL1 and NEUROD1 in DIPG**

Nichole A. Lewis^1, 2, 3^, Rachel Herndon Klein^1, 2, 3^, Cailin Kelly^1, 2, 3^, Jennifer Yee^1, 2, 3^ and Paul S. Knoepfler^1, 2, 3 *^

^1^Department of Cell Biology and Human Anatomy & ^2^Genome Center, University of California Davis School of Medicine; ^3^Institute of Pediatric Regenerative Medicine, Shriners Hospital For Children Northern California, Sacramento, CA 95817 *corresponding author, knoepfler@ucdavis.edu

- Supplementary Material -

**Figure S1. Genome-wide profile of accessible chromatin regions in H3.3K27M and H3.3-WT DIPG tumor samples. a.** Hierarchical clustering analysis of accessible chromatin regions via ATAC-seq comparing XIII XVII. **b.** Principal component analysis (PCA) of significantly differential peaks between all glioma lines.

**Table S1.** Summary of HOMER and DiffBind results for ATAC-seq samples.

**DIPG Cell Line ATAC-seq Analysis**

| **Sample** | **Peaks Counts** |
| --- | --- |
| XIII rep 1 | 64,889 |
| XIII rep 2 | 59,906 |
| XIII WT rep 1 | 68,076 |
| XIII WT rep 2 | 91,934 |
| XVII rep 1 | 51,520 |
| XVII rep 2 | 82,559 |
| XVII WT rep 1 | 70,331 |
| XVII WT rep 2 | 42,148 |

**DiffBind Analysis**

| **Sample** | **Unique Peaks** |
| --- | --- |
| XIII | 4,522 |
| XIII WT | 12,860 |
| XVII | 13,136 |
| XVII WT | 211 |

**Gene Region Peak Analysis**

| **Samples** | **Unique Peaks** | **Promoters** | **Gene Bodies** | **Intergenic Regions** | **Gene Bodies Excluding Promoter** | **Introns** | **Exons** | **Overlap with H3.3 ChIP-seq Peaks** |
| --- | --- | --- | --- | --- | --- | --- | --- | --- |
| XIII | 4,522 | 625 | 2,347 | 2,156 | 1,722 | 2,235 | 323 | 63 |
| XIII WT | 12,860 | 238 | 7,158 | 5,652 | 6,920 | 6,900 | 1,001 | 611 |
| XVII | 13,136 | 3,858 | 8,366 | 3,960 | 4,508 | 6,641 | 2,949 | 17 |
| XVII WT | 211 | 12 | 95 | 110 | 83 | 91 | 7 | 3 |

**DiffBind Analysis**

| **Sample** | **Shared Peaks** |
| --- | --- |
| XIII + XVII | 463 |
| XIII-WT + XVII-WT | 3,401 |
| XIII + XIII-WT | 41,190 |

**Figure S2. H3.3K27M DIPG cells are enriched for open chromatin peaks genes related to the nervous system and GTPase activity compared to their isogenic gene-edited wild-type counterparts.** Gene ontology analysis of differential open chromatin regions in both isogenic gene-edited wild-type cell lines. BP=Biological Processes, CC= Cellular Components, KEGG= KEGG Pathways, and MF= Molecular Function. For enriched GO terms p-values were obtained from the Benjamini-Hochberg method.

**Table S2.** Overlap of gene expression changes and ATAC-seq peak changes between WT and K27M cells.

| **Line XIII** | **K27M up genes** | **K27M down genes** |
| --- | --- | --- |
| XIII only ATAC-seq peaks | 1,881 | 1,215 |
| XIII-WT only ATAC-seq peaks | 1,814 | 4,874 |

| **Line XVII** | **K27M up genes** | **K27M down genes** |
| --- | --- | --- |
| XVII only ATAC-seq peaks | 5,627 | 1,758 |
| XVII-WT only ATAC-seq peaks | 41 | 119 |

**Table S3.** Overlap of enhancer and super-enhancer regions with ATAC-seq peaks in XIII and XVII cell lines.

| **Samples** | **Unique Peaks** | **Overlap with H3K27ac** | **Overlap with Enhancers** |
| --- | --- | --- | --- |
| XIII | 4,522 | 2,046 | 1,920 |
| XIII WT | 12,860 | 1,489 | 1,368 |
| XVII | 13,136 | 7,422 | 4,145 |
| XVII WT | 211 | 20 | 19 |

| **Samples** | **Unique Peaks** | **Overlap with Super-Enhancers** |
| --- | --- | --- |
| XIII | 4,522 | 306 |
| XIII WT | 12,860 | 292 |
| XVII | 13,136 | 309 |
| XVII WT | 211 | 2 |

**Figure S3. H3.3K27M DIPGs specific transcription factor binding sites are enriched in nervous system and neuronal development genes. a.** Transcription factor motifs identified as enriched via MEME-ChIP/FIMO and Homer. Percentage indicates increase of motifs in XIII and XVII, and **b** XIII-WT and XVII-WT cell lines over background. **c.** Gene ontology analysis of differential open chromatin regions with ASCL1 and, **d** NEUROD1 transcription factor binding motifs. For enriched GO terms p-values were obtained from the Benjamini-Hochberg method. **e.** HINT-ATAC line plots showing the differential footprints of transcription factors significantly differentially bound in XVII-WT (LHX6).

**Figure S4. Determination of enriched transcription factors shared between H3.3K27M and H3.3-WT peaks. a** Motifs identified using MEME-ChIP and number of times that motif occurred in the ATAC-seq peaks was determined using FIMO. Background was calculated by scrambling DNA sequence and inputting that sequence into FIMO. Threshold for significant fold enrichment was set to 2 (black dashed line). ***p-value<0.001 as described in MEME-ChIP output.

**Figure S5. H3.3K27M DIPG cells differentially express transcription factor ZEB2.** ZEB2 was selected for validation using qPCR. Fold change expression in H3.3K27M DIPG were calculated relative to H3.3-WT. n=3, **p-value<0.01, and error bars were calculated based on standard deviation.

**Table S4 A.** Table available in Supplementary_Tables_S4.xlsx file

**Table S4 B** Table available in Supplementary_Tables_S4.xlsx file
